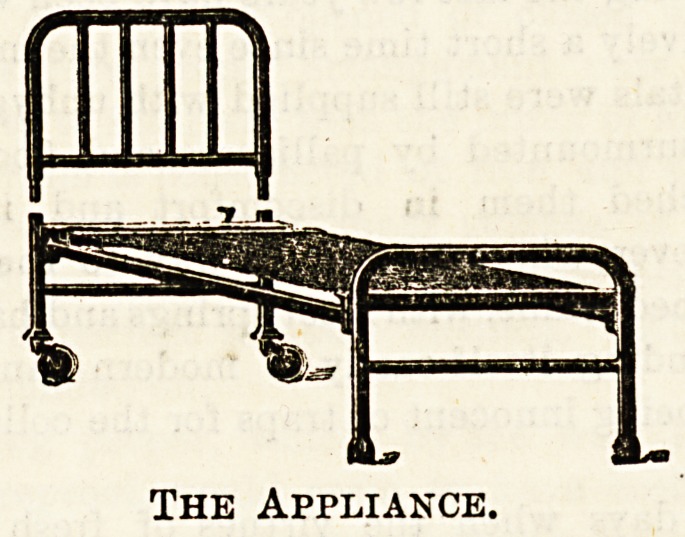# Institutional Furniture

**Published:** 1903-08-22

**Authors:** 


### INSTITUTIONAL FURNITURE.

### 1.—THE BEDSTEAD.

"THE hospital bed is the unit of the hospital." It is, therefore, the first and most important item when the furnishing and general equipment of a sick ward is under consideration, and must be given a front place when treating of institutional requirements. It goes without saying that simplicity of design and construction are the chief essentials which the hospital expert will endeavour to secure, combined with due regard for the comfort of the patient and the practical convenience of those who tend and nurse him. Doctors and nurses naturally have their own individual views as to the special points to be observed in selecting bedsteads for hospital use, and these will depend again upon the nature of the institution, whether hospital, convalescent home, asylum, or whatever it may be, and also upon the funds at disposal, the latter limitation being too often of a somewhat arbitrary nature. There are, however, one or two cardinal points which it is always essential to bear in mind when choosing beds for sick people, and it will be well to enumerate them before treating of any particular designs.

After the strength and durable qualities of the framework have been duly considered, there comes the question of height and width. For general hospital wards, where the patients may be all more or less looked upon as being seriously ill, the beds must be raised a good distance from the floor, to facilitate the nursing of helpless cases, which cannot, of course, be so easily managed upon a low bedstead. Experience has shown that a bed whose framework is less than 2 feet from the floor will entail a good deal of discomfort upon the nurse who is in charge, and a height of 2 feet 2 inches is none too high. It is a common complaint among hospital nurses that the beds are too low. For obvious reasons these beds should be no wider than is absolutely necessary for the patient's comfort, 2 feet 9 inches being a fair average width. On the other hand, for convalescent patients a lower bedstead, which is more easy to move freely about should be chosen. The foot-rail should be sufficiently raised above the mattress to prevent the occupant slipping down in the bed; the feeling of insecurity engendered by the lack of support is most trying to those who are weak and ill.

If there be a high foot-rail it should be detachable, to facilitate the work of the surgeon or anæsthetist in the event of an operation being necessary without moving the patient from bed. If the foot-rail be too low the application of an extension apparatus will be difficult. These points are, of course, more important in case of beds required for a surgical ward.

The improvements effected in the ordinary type of ward bedstead during the last few years have been very great. It is comparatively a short time since even the most up to date of our hospitals were still supplied with unhygienic wooden bedsteads, surmounted by palliasses and flock mattresses which matched them in discomfort and inconvenience. These have everywhere now given way to the cleanly and simple iron bed frame, with steel springs and hair mattresses, the whole lending itself easily to modern sanitary requirements, and being innocent of traps for the collection of dust and microbe.

In these days when the virtues of fresh air are more fully appreciated than they used to be, and when every well-constructed hospital has a balcony for each ward on to which patients' beds can be wheeled in fine weather, the question of castors is an important one. Whether fitted to the four legs or only to two the bed castors should be large—three to four inches in diameter—and preferably should have rubber tyres. With such tyres as these only can a bed be moved about easily and quietly and without jolting the patient.

Coming to details, where funds are not limited, or where it is realised that the truest economy is oftentimes most readily secured by the purchase of the best available article, there can be little question but that the pattern of bedstead with which the wards at Guy's Hospital are supplied throughout most nearly answers this description. A bedstead of this pattern, which is known as the Kirkland Patent Tubular bedstead, was originally designed by Dr. Perry, Medical Superintendent of that institution, and is certainly a most practical outcome of many years' hospital experience. It is severely plain in form, as hospital beds should be, and is made of steel tubes, which give the frame great strength, combined with light weight. Castors are usually fitted only to the legs at the head of the bed, while the other two are tipped with wooden or rubber feet. This plan prevents the bed slipping on a polished floor, while the nurse, by raising the foot can move it wherever required with perfect ease. The head piece is detachable; it can be entirely removed to allow a patient to be examined, anæsthetised, or operated upon, and when swung forward, as shown in the accompanying illustration, makes an excellent back-rest, these adjustments being carried out quite without exertion or difficulty. It will be noticed that the frame is entirely devoid of all unnecessary knobs and excrescences, often] seen in ordinary beds, and it should be remembered by all who are responsible for hospital furniture that these "ornaments," so-called, are utterly out of place whenever the dust fiend has to be fought with unsparing vigour, as in hospital. It is practically always needful to have a specially simple design carried out by whatever firm is selected to supply the beds. The makers of the Kirkland bedstead are Messrs. John Wilson and Co., 49 Lime Street, E.C. The price is, unfortunately, prohibitive, except

KIRKLAND PATENT TUBULAR BEDSTEAD.

INSTITUTIONAL BEDSTEAD.

under special circumstances, being between £5 and £6 per bed.

Beds of simpler construction than these may, of course, be procured at less cost, the ordinary solid iron frame being naturally a less expensive article to manufacture than a tubular one. Messrs. Heal, Tottenham Court Road, make a simple "Institutional Bedstead" at a very reasonable figure—from 25s. upwards—and have lately introduced a spiral spring mattress, which is intended as an improvement upon the more ordinary woven wire or chain spring mattresses, the springs, being equally distributed over the entire surface, having less tendency to give at any particular point.

The simplest form of hospital bed is that universally known as the "Lawson Tait," the makers of which are Messrs. G. Gale and Sons, Dominion Works, Birmingham. It is made in three parts only, is strong and cheap, and is the

Lawson Tait Bedstead.

bedstead very generally in use in hospital and asylum wards. One variation of this bed, here shown, has the elastic spring mattress divided near the head with a hinge, which allows the upper part to be raised to form a bed-rest. These "combination" bed-rests are to be preferred to those which are merely placed upon the bed, as they give a far firmer support and a better sense of rest to a sick person.

The Longford Wire Company, Warrington, have a deservedly good reputation for their hospital beds, which

The Carlton.

they are prepared to guarantee for five years. Their Carlton "three part-iron bedstead and wire mattress combined" is a good specimen of a plain institutional bed, and possesses the advantage, which should always be kept in mind, of removable head and end rails, giving facility for the administration of anæsthetics, and for operations. It also permits of the easy application of fracture boards and extension apparatus, points which have to be remembered when beds for hospital patients are under consideration. These beds are listed at £1 5s. upwards.

A good bedstead, which is very inexpensive, and of simple construction with perfectly smooth castings, is manufactured

Bedstead by Messrs. Evered & Co.

by Messrs. Evered and Co. (Surrey Works, Smethwick) with diamond spring mattress at 17s. and 18s. 3d.

The "Taunton" special hospital bedstead is another make which should be mentioned, designed and made by Messrs. John and Joseph Taunton, Limited, Belgrave Works, Birmingham. The "D-ag-nl" mattress as supplied by this

The Taunton.

firm is well known. The prices of these bedsteads are quoted from £2 13s. 9d. upwards, and a number of useful accessories in the way of lifting poles, irrigator poles, fracture boards, extension pulleys, etc., are made to fit where required.

Messrs. Isaac Chorlton and Co., Blackfriars Street, Manchester, are manufacturers on a large scale, showing very

The Appliance.

good designs. Their "Appliance" bedstead, with combined frame and back-rest is an admirable specimen of its kind, and we may also note for special approval the "Surgical" bedstead, with removable head and foot pieces, rubber tyres and feet, etc. It will be noticed that this and other designs of this firm are free from those undesirable and very ugly "knobs," before alluded to, and much to be commended on that account. The pattern supplied to the St. Mary's (New) Hospital, Manchester, is excellent, and so is that known as the "Patent Riveted Bedstead for Epileptics," with removable side rail. The prices run from £2 5s. for the plainest design upwards to £5 2s. 6d. for the "Appliance" bedstead.

One might continue this list almost *ad infinitum*, for there are great varieties of good institutional bedsteads on the market, but we have sufficiently indicated the lines upon which selection should be made, and details must naturally vary with individual requirements. The prices given are those at which the bedsteads are catalogued, but it will be, of course, understood that special quotations would be made in every case where any number is required.

## Figures and Tables

**Figure f1:**
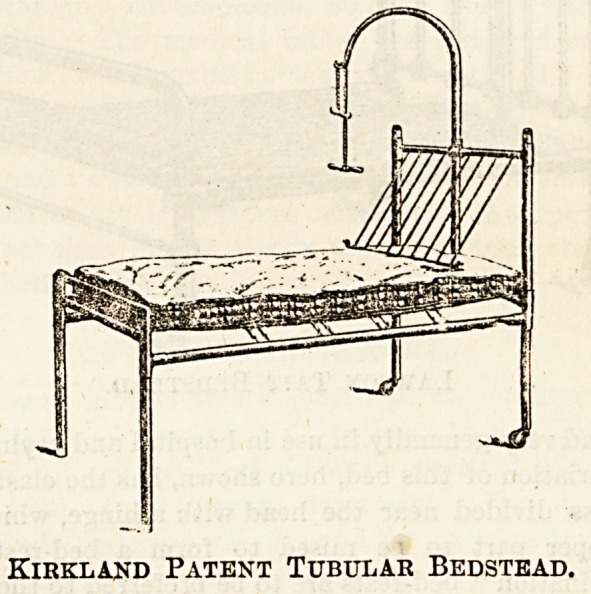


**Figure f2:**
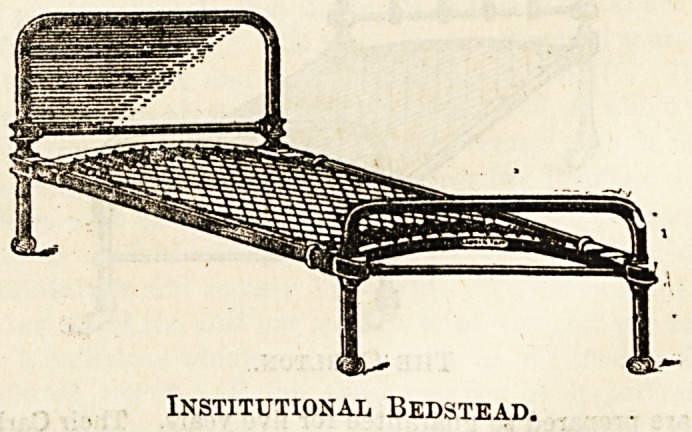


**Figure f3:**
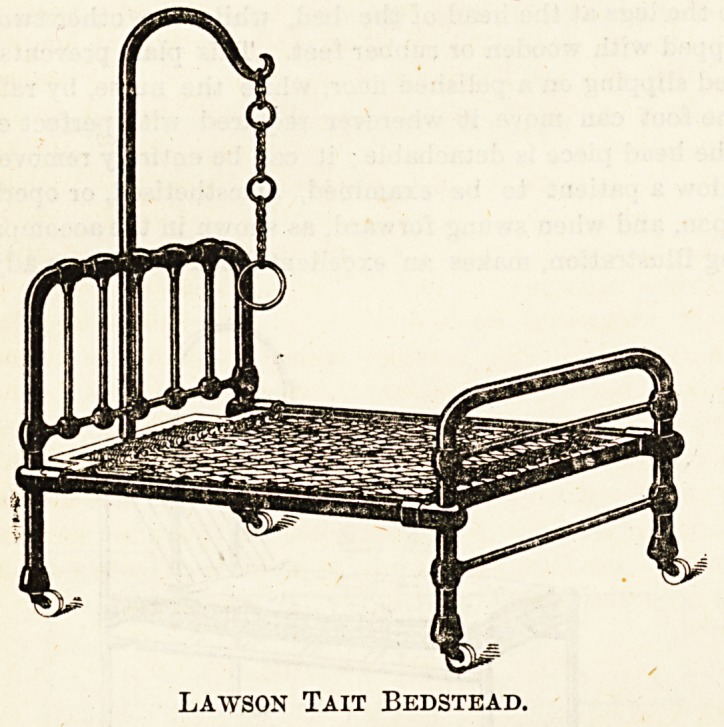


**Figure f4:**
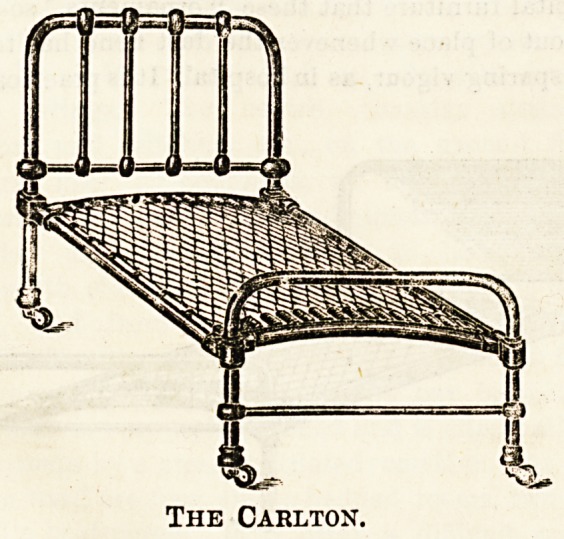


**Figure f5:**
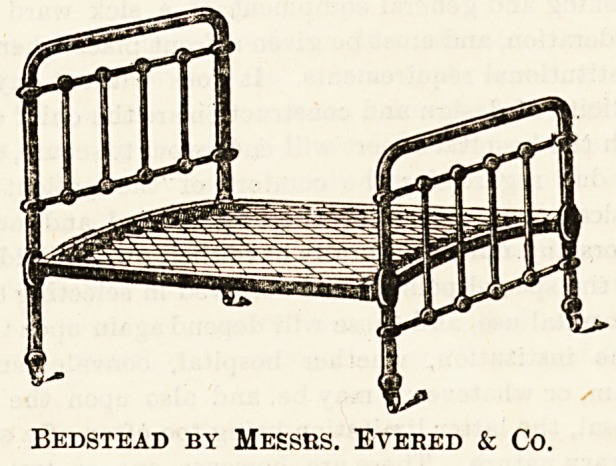


**Figure f6:**
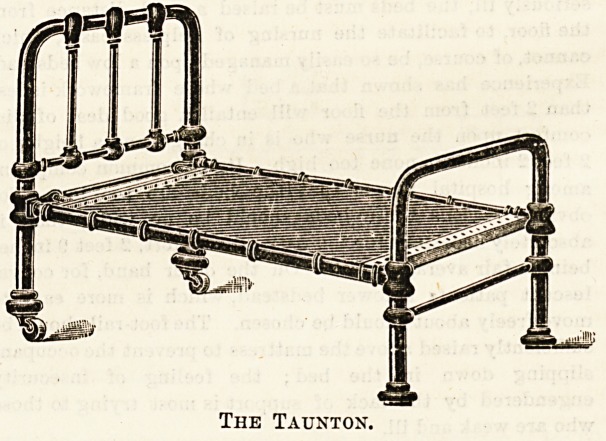


**Figure f7:**